# Sexual Health questions included in the Health Behaviour in School-aged Children (HBSC) Study: an international methodological pilot investigation

**DOI:** 10.1186/s12874-016-0270-8

**Published:** 2016-12-05

**Authors:** Honor Young, András Költő, Marta Reis, Elizabeth M. Saewyc, Nathalie Moreau, Lorraine Burke, Alina Cosma, Béat Windlin, Saoirse Nic Gabhainn, Emmanuelle Godeau

**Affiliations:** 1 Centre for the Development and Evaluation of Complex Interventions for Public Health Improvement (DECIPHer), School of Social Sciences, Cardiff University, Cardiff, UK; 2 National Institute of Health Promotion, Budapest, Hungary; 3 Eötvös Loránd University, Institute of Psychology, Budapest, Hungary; 4 Aventura Social - Faculdade de Motricidade Humana, Universidade de Lisboa, [University of Lisbon], Lisbon, Portugal; 5 ISAMB/Faculdade de Medicina da Universidade de Lisboa [Faculty of Medicine, University of Lisbon], Lisbon, Portugal; 6 FCT - Fundação para a Tecnologia e Ciência [Foundation for Science and Technology] (SFRH/BPD/110905/2015), Lisbon, Portugal; 7 University of British Columbia, School of Nursing, Vancouver, Canada; 8 Université Libre de Bruxelles (ULB), Service d’Information Promotion Education Santé (SIPES), School of Public Health, Brussels, Belgium; 9 Health Promotion Research Centre, National University of Ireland, Galway, Ireland; 10 Child and Adolescent Health Research Unit, School of Medicine, University of St Andrews, St Andrews, Scotland, UK; 11 Addiction Switzerland, Research Department, Lausanne, Switzerland; 12 Service Médical du Rectorat de l’académie de Toulouse, UMR 1027 Inserm, Université Paul Sabatier, Toulouse, France

**Keywords:** Adolescent sexual health, Adolescent sexual behaviour, Self-completion, Questionnaire design inconsistencies, Missing data

## Abstract

**Background:**

This paper describes the methodological developments of the sexual health items included in the Health Behaviour in School-aged Children (HBSC) study since their mandatory inclusion in the study in 2002. The current methodological, ethical and pedagogical challenges in measuring young people’s sexual health behaviours are discussed along with the issues associated with the sexual health items introduced to the HBSC study in 2002. The development and piloting of new cross-national items for use in the 2013/14 HBSC data collection are presented and discussed.

**Methods:**

An international pilot study was undertaken to determine the impact of these proposed changes. Questionnaires and classroom discussion groups were conducted in five pilot countries in 2012/2013 (France, Hungary, Ireland, Portugal and Romania) with a total of 612 school-aged children (age M = 15.55 years, SD = 0.95).

**Results:**

The majority of participants in each country provided positive feedback about the appropriateness of the questions. Some small cross-national differences were found in the self-reported quantitative data relating to the appropriateness of the questions (χ^2^ = 22.831, df = 9, *p* = .007, V = .117). Qualitative feedback suggests that for the vast majority of students the phrasing and age-targeting of the questions were considered appropriate. With the exception of a small number of respondents who commented on the clarity and/or personal nature of the content, no specific issues with the questions were identified.

**Conclusions:**

These findings provide guidance on the answerability (including the extent of missing and inconsistent data), understandability, acceptability (including in different cultures) and relevance of questions to potential participants. The findings from the pilot study suggest that in general, the questions are understandable, acceptable, and of a high priority to the target population, and that the simplification has significantly reduced the proportion of missing data. The new developments thus enhance the capacity of the questions to measure cross-nationally, sensitive aspects of young people’s sexual behaviour. These questions were included in the 2013/2014 round of the HBSC survey and will continue to be used to monitor trends in adolescent sexual health and behaviours, and to inform and influence health services and health education policy and practice at local, national and international levels.

## Background

The Health Behaviour in School-aged Children (HBSC) study is a cross-national research project carried out in collaboration with the World Health Organization (WHO). Founded in 1982, the study initially aimed to understand smoking behaviours in England, Finland and Norway. The HBSC study is now undertaken every four years in over 40 countries and regions across Europe and North America and has expanded to cover a wide range of indicators including health behaviours, risk behaviours and wellbeing [1]. The study also explores the social and developmental context in which young people live, along with their socio-economic conditions.

The study uses an anonymous, pen-and-paper, self-complete, internationally standardised questionnaire; administered in classrooms to gain the perspectives of a representative proportion of 11, 13 and 15 year old school-going children. In some countries, the protocol is also extended to other age groups. The study seeks to advance the scientific field of adolescent health internationally whilst acting as a monitoring tool to inform and influence health services and health education policy and practice at local, national and international levels [2].

### The importance of researching adolescent sexual health

Sexually active young people aged 15–24 years are at a greater risk of experiencing adverse health and social outcomes including unplanned pregnancy, early maternity, abortion or sexually transmitted infections (STIs) than older sexually active populations [3–5]. Early sexual initiation, inconsistent condom use and multiple sexual partners are recognised risk factors of STI transmission and unplanned pregnancy [6, 7]. Reproductive and sexual health, including the promotion of safe, healthy, positive sexual behaviour and reproductive choice, therefore remain a salient public health issue which warrants consistent monitoring and more detailed exploration [6, 8, 9]. Addressing the sexual health of young people by raising their commitment to safer sex has become a major issue among developed countries [10–12].

### The inclusion of sexual health questions in the HBSC Study

**Fig. 1:**
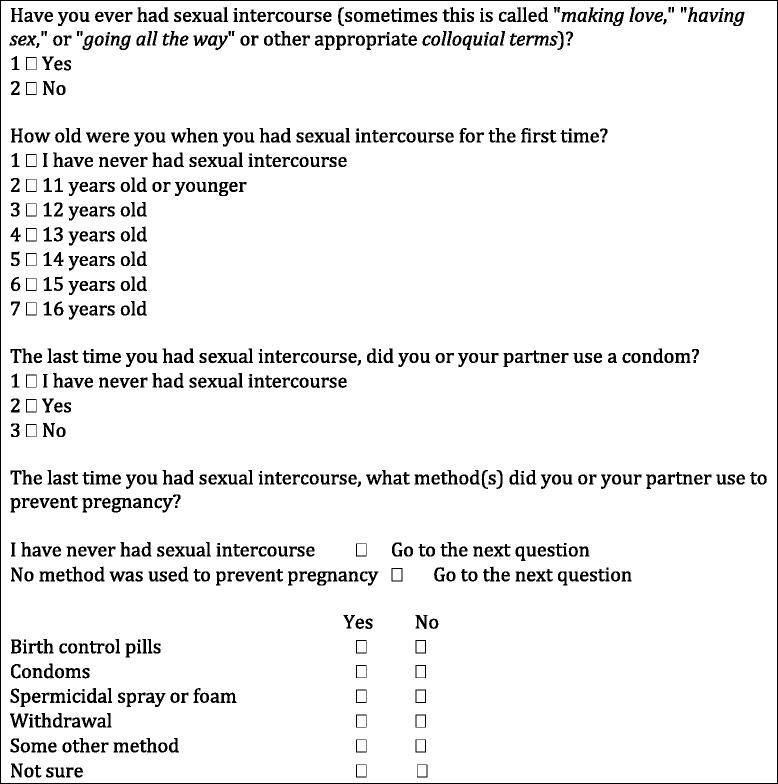
Standardised questions on sexual health in HBSC 2001/2002 survey

Few cross-national studies have been conducted to explore the sexual behaviour and contraceptive use of young people [13, 14], and rarely using comparable questions, methods or representative samples. Although the first questions relating to sexual health were included in the HBSC survey in 1989/1990, it was not until 2001/2002 that four standardised sexual health and behaviour questions were included. Derived from the US Youth Risk Behavior Surveillance study (YRBS) [15–17] and the 1986 Minnesota Adolescent Health survey [18], the questions measured experience of sexual intercourse, the age of sexual initiation, methods used to prevent pregnancy at last intercourse and condom use at last intercourse (Fig. 1). Two questions measured condom use, so as to represent two separate dimensions, one being pregnancy prevention and the other STI prevention.

The sexual health questions were designed to be mandatory (i.e., included in all countries or regions). However, each country or region was given the opportunity to opt-out of including these questions *in extremis* (i.e., if the sensitivity or inappropriateness of the items was likely to jeopardise the completion of the whole survey). In an effort to reduce such circumstances, the sexual health questions were recommended for administration only to 15-year-old students [19–24]. At this stage, 35 countries and regions included the sexual health items with only four refraining from using any of the questions; Denmark, Ireland, Norway and the United States. Data from Malta, Russia, Italy and the Czech Republic were excluded from analyses at this time due to deviations from the research protocol [21].

The 2005/2006 HBSC study included the same sexual health questions as those used in 2001/2002. Of the 41 participating countries, data were collected from 30 countries on experience of sexual intercourse, 31 on contraceptive pill use, and 30 on condom use [23, 25]. In the 2009/2010 HBSC survey, the same four questions were included as mandatory under the same conditions. Of the 41 participating countries, data were collected from 36 countries on experience of ever having sexual intercourse, 34 countries on contraceptive pill use, and 32 countries on condom use [24].

### Previous work using the HBSC sexual health items

The inclusion of standardised, mandatory sexual health questions in the HBSC surveys resulted in the publication of internationally comparable data on sexual initiation, [20, 22, 26], contraceptive use [23, 25, 27, 28], gender differences, sexual risk behaviour (e.g., non-contraceptive use), socio-economic inequalities and time trends analysis [24, 29]. Data from the HBSC study are not only used at an international level, but are also used to explore various aspects of young people’s sexual health and behaviour at a national level [30, 31]. These findings are of significant importance to professionals involved in the care of at-risk adolescents.

### Problems with the sexual health questions

Despite the inclusion of standardised, mandatory sexual health questions, considerable challenges remained in the interpretation of the international sexual health data. Some countries have introduced the use of skip patterns to avoid students completing questions which are not applicable. Although there are benefits associated, this has led to inconsistencies in the quality of the data collected.

Missing data currently poses the biggest issue for the collection of information about young people’s sexual health. Potentially sensitive, the questions are consequently only asked to participants aged 15 years and older. At the beginning of the questionnaire, participants are reminded that their responses are both anonymous and confidential, and they are encouraged to answer questions individually. Nevertheless, young people may refuse to answer the questions for reasons related to cultural norms, religious barriers, embarrassment or peer pressure, thus generating missing data. Many students who reported having engaged in sexual intercourse did not respond to subsequent questions relating to contraceptive methods also generating missing data. Further, the layout of the questions (Fig. 1) implies that participants who have not engaged in sexual intercourse had to report this four times, increasing the potential for frustration and stigmatisation.

In addition to the potential sensitivity of the questions, a substantial number of inconsistencies had been identified whereby respondents’ answers contradicted their responses in previous questions (e.g., reporting condom use, but also previously reporting that they had never had sexual intercourse). Similarly, some participants’ responses contradicted answers within the contraceptive use questions (e.g., reporting ‘no method of contraception was used’ and then reporting ‘yes’ to a subsequent form of contraception). Inconsistencies were also identified on the two separate questions addressing condom use (i.e., as a method to prevent pregnancy or as a method to prevent STI transmission).

Inconsistent, inaccurate, and missing data have posed difficulties for the comparison of data across HBSC survey rounds and across countries. Efforts to maintain continuity across countries and time periods have been made by the generation of an internationally comparable data file by the HBSC Data Management Centre. Countries who have asked questions that significantly deviate from the HBSC protocol are removed from the data set for those particular questions. There remained however some subjectivity about the further development and use of data at a country level, even if recoding syntaxes are centrally provided as guidance. This variability led to concerns about the reliability and comparability of these items.

In addition to these methodological issues, broader ethical and pedagogical issues have also been raised relating to the methods of contraception stated as pregnancy prevention. Concerns arose relating to the implied statement that withdrawal is an adequate method of pregnancy prevention. This issue may not be in isolation; in order to ensure the questionnaire is culturally relevant to young people, and to match some national programs, some countries add ‘national items’. Seven countries added ‘national items’ which included contraceptive methods considered inappropriate for this age group (e.g., withdrawal or natural/biological methods).

The issues associated with the sexual health questions across the past waves of the HBSC study prompted important changes to the instruments measuring sexual health and behaviour. The changes are presented below along with the findings from a cross-national pilot study of these proposed changes.

### Proposed changes to the sexual health questions

Three mandatory sexual health items are included in the 2013/2014 HBSC survey. To maintain comparability, these are largely similar to the mandatory questions from the 2001/2002, 2005/2006 and 2009/2010 surveys. The questions still measure participants’ experience of sexual intercourse, age at first intercourse, and the use of contraception at last sexual intercourse (condom, birth control pills and other methods) and are still only asked of students aged 15 years old and older. The main changes for the 2013/2014 international mandatory sexual health questions are the addition of a skip pattern and alterations to the layout of the contraceptive methods question.

**Fig. 2:**
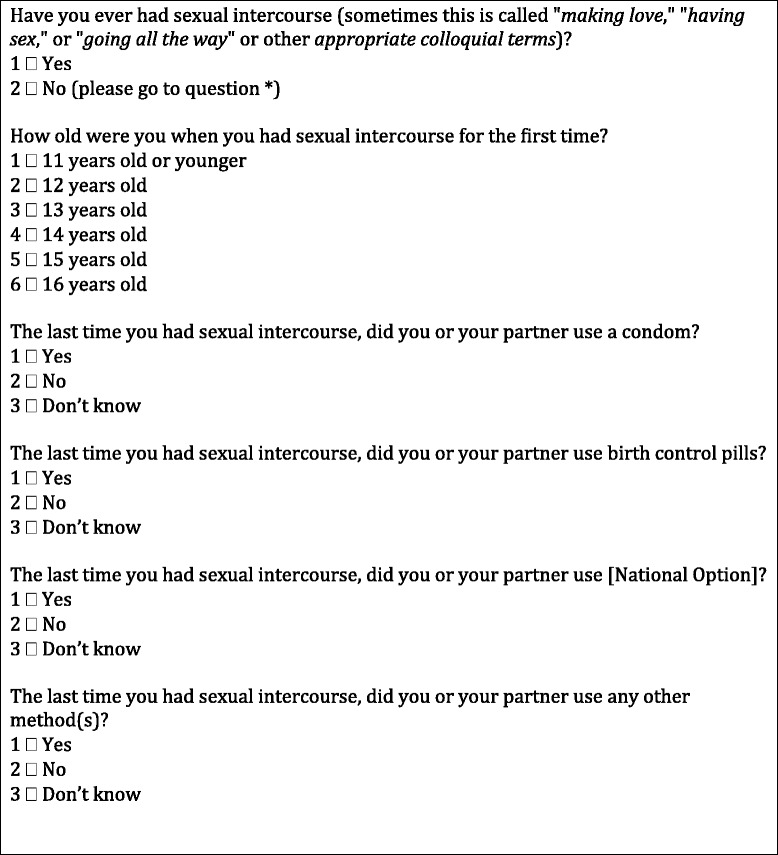
Standardised questions on sexual health in HBSC pilot survey

A skip pattern has been introduced after the first question “Have you ever had sexual intercourse (sometimes this is called “making love,” “having sex,” or “going all the way”)?” Respondents who have not had sexual intercourse are directed to the next applicable question. The response option “I have never had sexual intercourse” was removed from subsequent questions. This skip pattern simplifies coding, reduces inconsistencies across items, increases comparability between digital and offline surveys,^1^ and reduces stigmatisation and frustration of respondents who have not engaged in sexual intercourse. The second change was an alteration to the layout of the contraceptive methods question. To avoid the inconsistencies observed in earlier surveys, separate questions were developed to measure each method of contraception and one sole question measuring condom use was introduced (Fig. 2). Finally, in order to overcome ethical and pedagogical limitations, the question wording was altered to remove the connotations that national options such as ‘withdrawal’ were a form of pregnancy prevention. Instead, the questions read “The last time you had sexual intercourse, did you or your partner use” followed by the given method.

## Aim

The aim of the pilot was to provide guidance to the international network about the following dimensions of the proposed items; answerability (including the extent of missing and inconsistent data), understandability, acceptability (including in different cultures and both genders) and relevance to potential participants. The findings have been used to guide the inclusion of items for the 2013/2014 HBSC study.

## Methods

### Pilot study of proposed changes

A pilot of the proposed new mandatory items was conducted between 2012 and 2013 in five countries; France (*n* = 76), Hungary (*n* = 188), Ireland (*n* = 228), Portugal (*n* = 30) and Romania (*n* = 90), representing a wide range of cultural backgrounds. Ethical approval was sought from the University Ethics Boards or other authorities associated with the research team in each country. Schools were recruited based on a purposive convenience sampling method to reflect a range of public, private, rural and urban schools, and those with varying levels of family and socio-economic backgrounds. Class selection was left at the discretion of the school, based on the age range of participants required for the pilot study. All young people in the selected classes were invited to participate. Table 1 illustrates the characteristics and distribution of schools and classes recruited within each country. Finally, 612 students aged between 15–17 years (mean age = 15.55 years, median age = 16 years, SD = 0.95) from public, private, rural and urban schools from five countries were involved in the pilot.

**Table 1:** The distribution and characteristics of schools and classes recruited to take part in the pilot study within each country

		Number of respondents in class (Class n)					
		1	2	3	4	5	6
France Mean = 14.93, Median = 15.00, SD = 1.08 F = 59.2%, n = 45	School 1 Urban public junior high school Mean = 15, Median = 15, SD = .000, F = 66.7%, n = 8	12					
	School 2 Urban private vocational high school Mean = 16.62, Median = 17 SD = .506, F = 38.5, n = 5	9	4				
	School 3 Rural public junior high school Mean = 14.49, Median = 14 SD = .857, F = 62.7, n = 32	15	19	17			
Hungary Mean = 15.77, Median = 15.00, SD = .92 F = 56.4%, n = 106	School 1 Suburban public high school Mean = 15.77, Median = 15 SD = .924, F = 56.4, n = 106	28	33	33	32	28	34
Ireland Mean = 15.92, Median = 16.00, SD = .55 F = 37.7%, n = 86	School 1 Urban mixed gender school Mean = 16.59, Median = 17 SD = .568, F = 48.3, n = 14	18	11				
	School 2 Urban boys school Mean = 15.85, Median = 16 SD = .428, F = 0, n = 0	14	23	20	19	22	19
	School 3 Urban girls school Mean = 15.82, Median = 16 SD = .459, F = 100, n = 34	18	16				
	School 4 Urban girls school Mean = 15.65, Median = 16 SD = .485, F = 100, n = 34	34					
	School 5 Urban mixed vocational school Mean = 16.07, Median = 16 SD = .730, F = 28.6, n = 4	14					
Portugal Data not available	School 1 Urban public high school	30					
Romania Mean = 14.69, Mean = 15.00, SD = .53 F = 58.9%, n = 53	School 1 Urban public high school Mean = 14.69, Median = 15 SD = .533, F = 58.9, n = 53	11	26	29	24		

Parental consent was obtained based on the discretion of the school (active or passive depending on each country’s school policies). All participants were asked to provide active informed consent prior to participation. The mixed methods pilot study involved a questionnaire administered to students followed by a qualitative classroom exploration. All questionnaires contained the proposed items relating to experience of sexual intercourse, contraception use at last intercourse and age at first intercourse. Additional questions relating to sexual behaviour were asked during the piloting process. These included experience of romantic relationships, use of contraception at first intercourse, age of partner at first intercourse, use of alcohol/other substances at first intercourse and perception of timing of first intercourse (i.e., did you want it to happen at that time?). All questions underwent a translation and back-translation process, in accordance with the HBSC international protocol, to ensure international consistency and comparability. In Ireland and France, additional questions on bullying, alcohol use behaviour and self-harm were also piloted. All participants were asked socio-demographic questions. At the end of the questionnaire participants were asked “when you filled in the questions, did you think…?”, with response options “none,” “some,” “most,” or “all of the questions are inappropriate for young people your age.” An open-text feedback box at the end of the questionnaire was also provided, reading “In the box below you can say anything you like about these questions. Tell us if you understood the questions or if you feel they are okay to ask young people your age. Remember it is totally anonymous and nobody except the researchers will see what you write.” Prior to filling in the questionnaire participants were assured that they did not have to answer any questions that they were uncomfortable with and were encouraged to highlight this issue in their feedback if it was the case.

Based on shared guidelines, qualitative data collection followed the individual completion of the questionnaire. Participants were first asked to underline any words/phrases which were difficult to understand and to provide written feedback about each item and/or items they found problematic or inappropriate. Subsequently participants were invited to participate in a collective class discussion. Participants were first given a chance to have an open discussion about the questionnaire. They were then asked to discuss whether they felt that the overall topic was acceptable, relevant and appropriate to young people their age. Each item was then considered individually, and young people were asked open response questions as to whether they felt the questions were appropriate, relevant and what was able to be understood by young people their age. Quantitative data were collected from France, Hungary, Ireland and Romania (not Portugal) with a total of 582 participants. Qualitative data were collected from all countries with a total of 612 participants.

## Results

### Answerability

When asked if they had ever engaged in sexual intercourse, 19.2% of the participants (*n* = 112) indicated “yes”. A small proportion (*n* = 19; 3.3%) did not provide an answer to this question.

Table 2 illustrates the response rates and missing values for reported contraception use at last intercourse among young people who reported having had sexual intercourse. Response rates were very high; prevalence of missing values ranged from 2.7 to 6.3%. No significant gender or country level differences were identified in the proportion of respondents or missing data for any of the contraceptive questions.

**Table 2:** Response rate and missing values for contraception at last intercourse among young people who reported having had sexual intercourse (*n* = 112)

	Respondents % (n)	Missing % (n)
Condom	97.3 (109)	2.7 (3)
Pill	95.5 (107)	4.5 (5)
Other method	93.8 (105)	6.3 (7)

Table 3 illustrates the reported contraceptive use at last intercourse among the young people who reported having had sexual intercourse. Of those participants who reported having sexual intercourse and answered the question on condom use (*n* = 109), 66.1% reported condom use, 28.6% reported that a condom was not used and 2.7% did not know whether they or their partner used a condom at last intercourse. Three participants who reported sexual initiation (2.7%) did not answer this question.

**Table 3:** Contraception use at last intercourse among the young people who reported having had sexual intercourse

	Use % (n)	No use % (n)	Do not know % (n)
Condom (*n* = 109)	66.1 (74)	28.6 (32)	2.7 (3)
Pill (*n* = 107)	17.0 (19)	62.5 (70)	16.1 (18)
Other method (*n* = 105)	9.8 (11)	67.0 (75)	17.0 (19)

Of the participants who reported experience of sexual intercourse and answered the question on birth control pill use (*n* = 107), 17% reported contraceptive pill use at last intercourse, 62.5% reported no use of birth control pills and 16.1% of respondents did not know if birth control pills were used at last intercourse. Five participants who reported sexual initiation (4.5%) did not answer this question.

Of those participants who reported experience of sexual intercourse and answered the question on other contraceptive methods use (*n* = 105), 9.8% reported some other method of contraception at last intercourse, 67.0% reported no other method of contraception was used, and 17.0% did not know if another method of contraception was used at last intercourse. Seven participants who reported sexual initiation (6.3%) did not respond to this question.

Of those respondents who reported sexual initiation, 96.4% (*n* = 108) reported the age at which they first had intercourse. Four participants who reported sexual initiation (3.6%) did not respond to this question.

Despite the skip pattern, some participants gave inconsistent responses; of those participants who reported never having engaged in sexual intercourse, two (0.4%) reported “using a condom at last intercourse,” three (0.7%) reported “don’t know” in relation to condom use and three (0.7%) reported “don’t know” in relation to the use of other contraceptive methods at last intercourse. One participant (0.2%) reported using a condom at last intercourse, despite leaving the question on experience of sexual intercourse blank. One participant (0.2%) reported using the contraceptive pill while reporting never having engaged in intercourse. According to the skip logic, this may appear inconsistent; however it is possible that this participant was indeed on the contraceptive pill without having engaged in sexual intercourse. Finally, three participants (0.7%) reported that they did not know if birth control pills were used at last intercourse, while saying that they did not have sexual intercourse. No participants who reported never having engaged in sexual intercourse reported an age of first intercourse (i.e., inconsistent data).

### Understandability

During the classroom discussion the overwhelming majority of students understood the terms ‘sexual intercourse’ and the colloquial terms used. Students generally reported no problems understanding questions relating to contraceptive methods or the age of first intercourse. When asked to provide “general comments” in the open text box at the end of the questionnaire, 219 respondents (37.6%) provided responses. A total of 174 respondents (79.4%) recorded that the questions were clear and/or easy to understand.

*“All the questions are clear, do not change a thing” (Participant, Hungary)*

*“This questionnaire is very easy to get” (Participant, France)*

*“Easily understood and straightforward” (Participant, Ireland)*

Only five respondents (2.3%) reported the questions as unclear or confusing.

*“I found the ‘now skip to question…’ a bit confusing. The questions are okay” (Participant, Ireland)*

*“Sometimes a bit confusing to follow.” (Participant, Ireland).*

Although the skip logic reduced the proportion of missing and inconsistent data, skip patterns in general can create some challenges in surveys of adolescents. In this pilot, the small number of participants who expressed confusion about the items indicated that the skip pattern instructions were the unclear aspect.

### Acceptability of sexual behaviour questions and relevance to participants

Of the 559 respondents to the question relating to the inappropriateness of the questions, 64.3% reported that none of the questions were inappropriate while 19.6% reported that some of the questions were inappropriate. A total of 7.4% reported that most of the questions were inappropriate, whereas 4.5% reported that all of the questions were inappropriate. Chi-square tests identified significant cross-national differences in the responses (χ^2^ = 22.831, df = 9, *p* = .007). Young people across all four regions were similar in the proportion of those reporting that “some” or “all” of the questions are age-inappropriate. Fewer Romanian participants indicated that they found “none” of the questions inappropriate, whereas fewer Hungarian respondents reported that “most” of the items were inappropriate. Cramér’s *V* was used to provide an estimate of the effect size of the association, *V* = .117, *p* = .007. This indicates that despite the significant association between region and ratings of age-inappropriateness, it is a very low effect. No significant association was found between the acceptability of the questions and age (χ^2^ = 5.967, df = 9, *p* = .743) or gender of participants (χ^2^ = 2.132, df = 3, *p* = .545). Cross-cultural or even school-level differences may confound the association, and the results should be treated with caution due to the low sample sizes. Given that additional questions relating to sexual behaviour were asked during the piloting process, and that in Ireland and France, additional questions on other health behaviours were also included, it is important to acknowledge that comments relating to the appropriateness of questions may relate to these supplementary questions on the questionnaire, even when the researchers facilitating the focus group discussion were asked to clearly separate the topics.

When reviewing the data from the written comments on the questionnaires, the main concern expressed about the question asking whether participants had ever engaged in sexual intercourse (*n* = 3) was that the question was limited to penetrative sexual intercourse.

*“Most people our age have done everything but sex, just saying” (Participant, Ireland)*

One participant questioned the usefulness of the explanation between the brackets. This was also raised by two students in the class discussion in Portugal. Aside from these comments, students did not raise written concerns about the acceptability and relevance of this question. Similarly, no specific comments relating to acceptability or relevance were volunteered about the contraceptive use or the age of first intercourse questions. During the class discussion some students expressed embarrassment, and a small minority considered the questions private, however there were no significant objections to the questions, or reports that they were unacceptable or irrelevant to young people this age.

Of the 219 participants who provided responses to the “general comments” section at the end of the questionnaire, seven said that the questions were private or personal, but did not specify whether this was a problem. A total of 25 students stated that the questions were inappropriate, too personal or private or felt that they were not adapted to people of their age group (France, *n* = 1; Hungary, *n* = 7; Ireland, *n* = 14; Romania, *n* = 3).

*“I feel that some of the questions were quite invasive and too personal.” (Participant, Ireland)*

*“I want to say that some items are too private and they should not be used because they refer to your private life. Some students are shyer and they don’t talk about their life.” (Participant, Romania)*

*“The questions are clear but sometimes childish; teenagers in my age can talk about sex seriously.” (Participant, Hungary)*

A total of 178 students reported that they felt the questions were appropriate, suitable for their age or relevant to young people.

*“All the questions are clear; you can ask my peers about the topic” (Participant, Hungary)*

*“These questions were perfect to present to teenagers. They tackle all important issues in a teenagers life, and they were really well laid out and clear” (Participant, Ireland)*

*“Your questionnaire is well done, adapted to the teenagers” (Participant, France)*

A small number of students (*n* = 13) raised concerns about whether students would answer honestly.

*“Some people are not going to say if they did some of the things mentioned” (Participant, Ireland)*

*“The questions are clear, but I don’t think everyone answered honestly” (Participant, Hungary)*

A total of 28 participants provided suggestions for additional, more detailed sexual health/behaviour questions that they felt would also be important to young people (e.g., relating to virginity, wanting to have sex, other sexual behaviours and different types of relationships).

*“It could be more profound, and should refer to topics such as sex education or petting” (Participant, Hungary).*

*“Should have more questions for virgins” (Participant, Hungary)*

*“Should include more about boy-girl friendships” (Participant, Ireland)*

### Reliability

A test-retest study was carried out in three schools in Ireland consisting of a total of 63 participants. Kappa statistics were calculated for items measuring engagement in sexual intercourse (κ = 0.832), age of sexual initiation (κ = 0.788) and condom (κ = 0.730), birth control pill (κ = 0.781) and other contraceptive (κ = 0.451) use at last intercourse. Apart from the question on other contraceptive used at last intercourse, all contained a sufficient level of inter-rater reliability (i.e., kappa above 0.6) indicating their suitability for use in the HBSC survey.

## Discussion

An international pilot study conducted in five countries suggests that despite some small cross-national differences in the self-reported quantitative data relating to the appropriateness of the questions, the majority of participants in each country reported positive feedback about the appropriateness of the questions. Qualitative feedback suggests that, overall, the phrasing and age-targeting of the questions were considered suitable by the vast majority of students. With the exception of a small number of respondents who commented on the clarity and/or personal nature of the content, no specific issues with the questions were identified.

Missing and inconsistent data posed two of the most significant issues with previous HBSC survey rounds. The newly introduced skip pattern and alterations to the layout of the contraceptive methods question have reduced the potential for participants to provide missing and inconsistent data. When comparing the consistent, inconsistent and missing responses from the 2009/10 HBSC study data (limited to the four target countries) and the 2013-pilot data, the proportion of the students who responded in an inconsistent way has reduced, although not significantly across the study sweeps (1.4% in 2010 and 1.0% in the 2013 pilot). However, simplification of the item structure resulted in a significant decrease in the number of missing responses, from 6.5 to 3.1% (χ^2^ = 29.651, df = 3, *p* < .001). This positively impacts on data quality for future HBSC survey rounds of data collection. Some countries have also introduced digital questionnaire completion. This would further reduce the problem of inconsistent responses, preventing those who do not report having had sexual intercourse from spending time on any related questions. Online testing would also simplify the skip question instructions mentioned by some as a problem. This benefit would go beyond the sexual health questions if introduced as a future HBSC standard.

For practical, political and ethical reasons, there has been difficulty including the sexual health questions in the HBSC study in some countries. The current findings indicate that young people from five culturally different countries, on the whole, are open to answering anonymous questions relating to their sexual behaviour. The vast majority of feedback, in relation to acceptability and relevance of these questions, was positive. Even in those countries where the sexual health questions are included in the HBSC survey, asking young people questions about sexual health and behaviour may be potentially embarrassing or even annoying. For this reason, it is crucial to strive for the most simple and neutral question format. As a result of the pilot study feedback, changes to the pilot have ensured that participants are reminded that they can choose not to answer any questions that they do not want to answer.

Safe and healthy sexual behaviour, including reproductive choice, not only play a key role in young people’s health and wellbeing, but are also salient public health issues. With many young people initiating sexual intercourse and engaged in risk behaviours during adolescence, it is a crucial time to measure young people’s sexual behaviours as a way to inform and influence health services, and health education policy and practice at local, national and international levels. There is currently very limited high-quality, cross-national data available which explores the sexual behaviour and contraceptive use of young people. The HBSC study therefore provides a unique opportunity to collect high quality, internationally comparable data not only about the sexual health behaviours of young people, but also about the context in which young people live and develop.

Methodological, ethical and pedagogical issues had however been raised relating to the sexual health items introduction on a mandatory basis to the HBSC study in 2002. These issues have prompted important changes to the questions measuring sexual behaviour; the addition of a skip pattern and alterations to the layout of the contraceptive methods question. The results have provided guidance on the answerability (including the extent of missing and inconsistent data), understandability, acceptability (including in different cultures) and relevance to potential participants.

The pilot study is not however without limitations. First it should be acknowledged that the sample size is quite small and not representative at country level, despite an effort to collect data in contrasting schools. Completion of the questionnaire required self-report data from young people about the potentially sensitive topic of sexual behaviour. Although participants completed questionnaires both anonymously and confidentially, and every effort was made to ensure that young people completed the questionnaire individually (i.e., without any influence from their peers), the limitations of collecting self-report data about sensitive topics has been well documented [32]. In addition, whilst participants were given the opportunity to provide written feedback about their acceptability and understanding of the questions, they may have been unwilling to disclose their lack of understanding or embarrassment of the questions verbally in the non-anonymous peer-group group discussion.

Another limitation is the current wording of the question relating to sexual intercourse. It has been shown that with the help of the information given in parenthesis, young people interpret this as penetrative vaginal sex only [33, 34], however no anatomical definition of sexual intercourse is provided. This also leaves aside other forms of sexual behaviour that could be an STI risk. Additional questions were included in the pilot study that looked at experience of romantic relationships, including a question that explored same-sex relationships. It is hoped that the inclusion of these questions would prevent young people from feeling marginalised by any heteronormative questions, and although they may not report feelings of marginalisation in the class discussion, any comments could be made when instructed to write on the questionnaire.

Finally, it must also be acknowledged that the pilot questionnaire contained additional questions relating to romantic relationships and the circumstances surrounding first sexual intercourse (contraception, age of partner, use of substances), and in Ireland and France, further additional questions on bullying, alcohol use and self-harm. The evaluation of the sexual health items may also partially reflect an evaluation of these questions or be altered by the evaluation of these questions.

## Conclusion

To summarise, following the issues highlighted in relation to the mandatory sexual health questions included in the HBSC survey since 2002, changes have been made to the HBSC survey questions. The core meaning of the questions has not changed, but findings from the pilot study suggest that the simplification has significantly reduced the proportion of missing data, thus enhancing the capacity of the questions to measure sensitive aspects of young people’s sexual behaviour cross-nationally. As such the adapted questions have been included in the 2013/2014 round of the HBSC survey. The Sexual Health Focus Group within the HBSC Research Network will continue to monitor trends in adolescent sexual health and behaviours and improve their measures, with the goal to inform and influence health services, decision makers in health education policy and all professionals caring for adolescents at local, national and international levels.

## Abbreviations

HBSC: Health behaviour in school-aged children
HIV: Human immunodeficiency virus
STI: Sexually transmitted infection
STIs: Sexually transmitted infections

## Footnotes

1 A number of countries had already introduced skip patterns into their questions, but this has not been applied consistently across the international methodological design.
